# Data of a fluorescent imaging-based analysis of anti-cancer drug effects on three-dimensional cultures of breast cancer cells

**DOI:** 10.1016/j.dib.2015.09.037

**Published:** 2015-10-08

**Authors:** Junji Itou, Sunao Tanaka, Wenzhao Li, Yoshiaki Matsumoto, Fumiaki Sato, Masakazu Toi

**Affiliations:** Department of Breast Surgery, Graduate School of Medicine, Kyoto University, 54 Shogoin-Kawahara-cho, Sakyo-ku, Kyoto 606-8507, Japan

**Keywords:** Anti-cancer drug, Breast cancer, Live imaging, Photoconvertible fluorescent protein, Pulse labeling, Three-dimensional culture

## Abstract

Three-dimensional (3D) cell culture is a powerful tool to study cell growth under 3D condition. To perform a simple test for anti-cancer drugs in 3D culture, visualization of non-proliferated cells is required. We propose a fluorescent imaging-based assay to analyze cancer cell proliferation in 3D culture. We used a pulse-labeling technique with a photoconvertible fluorescent protein Kaede to identify non-proliferated cells. This assay allows us to observe change in cell proliferation in 3D culture by simple imaging. Using this assay, we obtained the data of the effects of anti-cancer drugs, 5-fluorouracil and PD0332991 in a breast cancer cell line, MCF-7.

**Specifications table**

**Table:** 

Subject area	Biology
More specific subject area	Cell biology, drug development
Type of data	Image, graph, figure
How data was acquired	Microscope
Data format	Analyzed
Experimental factors	Breast cancer cells were cultured in three-dimensional condition.
Experimental features	Cells were pulse-labeled with Kaede-red fluorescent protein, and analyzed whether cells were proliferated. The effects of anti-cancer drugs were tested.
Data source location	Kyoto, Japan
Data accessibility	Data is in this article. The detailed procedure is in the supplementary material.

## 1 Value of the data

• Pulse-labeling with fluorescent protein is useful technique to analyze cell proliferation in three-dimensional culture.
• Non-proliferated cells are easily identified by pulse-labeling in three-dimensional culture.
• Our fluorescent imaging-based analysis can evaluate anti-cancer drug effects on cell proliferation.

## 2 Data, experimental design, materials and methods

In the process of drug development, animal studies are required to evaluate candidates obtained from two-dimensional (2D) cell culture. However, experiments with animals are costly and labor-intensive, and should be reduced to protect animals. Therefore, a useful pre-animal study model is in demand. Three-dimensional (3D) culture is considered to have more similar characteristics to the in vivo environment than to 2D culture, and is a favorable technique to fill the gap between 2D cultures and animal studies.

The desired function of most anti-cancer drugs is proliferative inhibition. In 3D culture, change in cancer growth is analyzed by observation of the size and the morphology of a colony, and it is difficult to judge whether alterations are due to changes in cell proliferation and/or survival. To overcome this problem, simple proliferation assay is needed.

We demonstrate a fluorescent imaging-based assay to analyze anti-cancer drug effects on 3D growth. We utilized a fluorescent protein Kaede for pulse-labeling. The fluorescent color of Kaede can be irreversibly changed from green to red (Kaede-red) by irradiation with short wavelength light [1]. We used a luminal breast cancer cell line, MCF-7, and performed pulse-labeling with Kaede-red for visualization of non-proliferated cells. Cells were treated with anti-cancer drugs, and subsequent changes in cell proliferation were analyzed.

### 2.1 Material and methods

A detailed procedure for the fluorescent imaging-based assay is described in the supplementary material.

Establishment and maintenance of Kaede-expressing MCF-7 cells were described previously [2]. For 3D culture, cells were suspended in 5% phenol red-free Matrigel (Corning, 356237, Bedford, MA, USA) and plated in a Matrigel-coated well of a clear-bottom 96-well plate (BD Falcon, 353219, Franklin Lakes, NJ, USA). Anti-cancer drugs we used were 5-fluorouracil (Wako, 064-01403, Osaka, Japan) and PD0332991 (Selleck Chemicals, S1116, Houston, TX, USA).

Images were collected with an all-in-one microscope, BZ-9000 (Keyence, Osaka, Japan). To generate Kaede-red, cultures were irradiated with 340–380 nm light for 2.5 minutes. Twelve sections were taken every 5 μm, and stacked using BZ-II software (Keyence). The size of each picture is 1.45 mm×1.04 mm. Kaede-red intensity was obtained by measuring the mean value of each nucleus using ImageJ software.

Statistical analyses were performed with Student׳s *t*-test. *P*<0.05 was considered statistically significant.

### 2.2 Pulse-labeling with Kaede-red

**Fig. 1:**
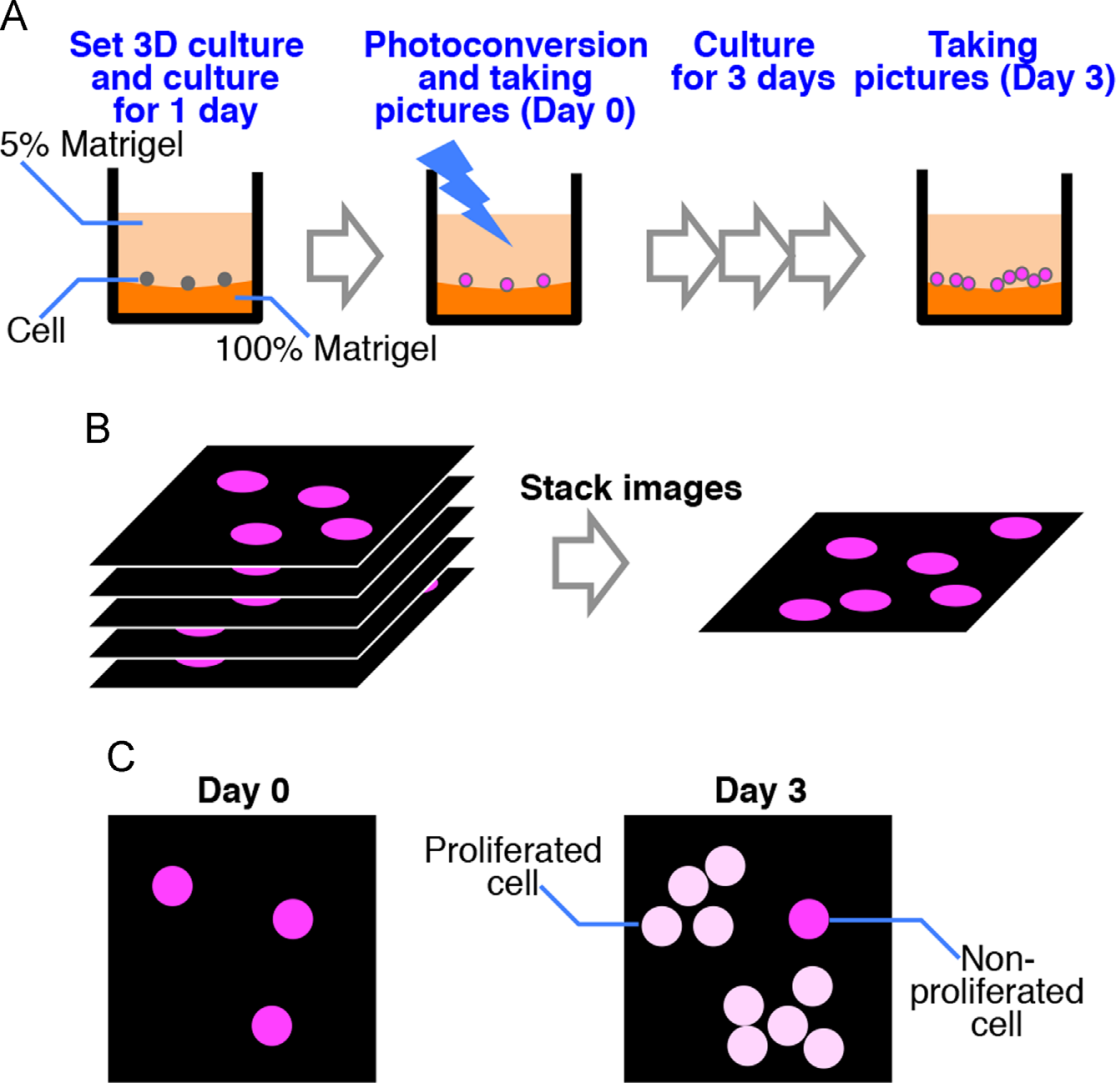
A fluorescent imaging method for analyses of cell proliferation under 3D condition. (A) Cartoon of 3D culturing and pulse-labeling with Kaede-red. Cells were cultured on a basement membrane matrix and irradiated with short wavelength light for photoconversion. (B) Cartoon of image acquisition and focus stacking. (C) Fluorescent imaging to detect proliferated, and non-proliferated cells. At day 0, all cells have strong Kaede-red. At day 3, proliferated cells have weak Kaede-red signals. Non-proliferated cells are identified as cells with strong Kaede-red signals.

Previously we established Kaede-expressing cell lines that have Kaede expression in their nuclei, and optimized a non-toxic photoconversion condition for pulse-labeling [2]. The Kaede-expressing cells were cultured in a 3D culture system on the basement membrane matrix (Fig. 1A). In high-density 3D culture, signals overlap. To avoid overlapping fluorescent signals, we plated small numbers of cells (e.g. 4×10^4^ MCF-7 cells was used per well). One day after setting up the 3D culture, cells were irradiated with 340–380 nm light to generate Kaede-red.

To analyze signals from the 3D structure, serial *z*-axis sections were collected and merged to form an image via focus stacking (Fig. 1B). We took pictures on day 0 and day 3 with the same exposure time to determine whether Kaede-red intensity was reduced at day 3.

Kaede-red molecules are generated only when a cell is exposed to short wavelength light, and the intensity of Kaede-red fluorescence per cell is reduced by cell division [2]. Therefore, proliferated cells have weak Kaede-red fluorescence after culturing, while cells that have not proliferated exhibit strong fluorescence (Fig. 1C).

### 2.3 Treatment with 5-fluorouracil and PD0332991

Our assay enables us to analyze changes in the number of non-proliferated cells after drug administration. To analyze the effect of a drug, we added the drug after taking pictures at day 0. Subsequently, cells were cultured for 3 days, and pictures were taken.

We calculated the mean value of Kaede-red at day 0, which was used as a reference to analyze the change in Kaede-red intensities of the cells in the corresponding day 3 culture. We defined cells having a Kaede-red intensity equal to or stronger than 80% of the mean value of day 0 as non-proliferated cells at day 3, because our previous study has shown that Kaede-red intensity is reduced to less than 78% by cell division [2].

**Fig. 2:**
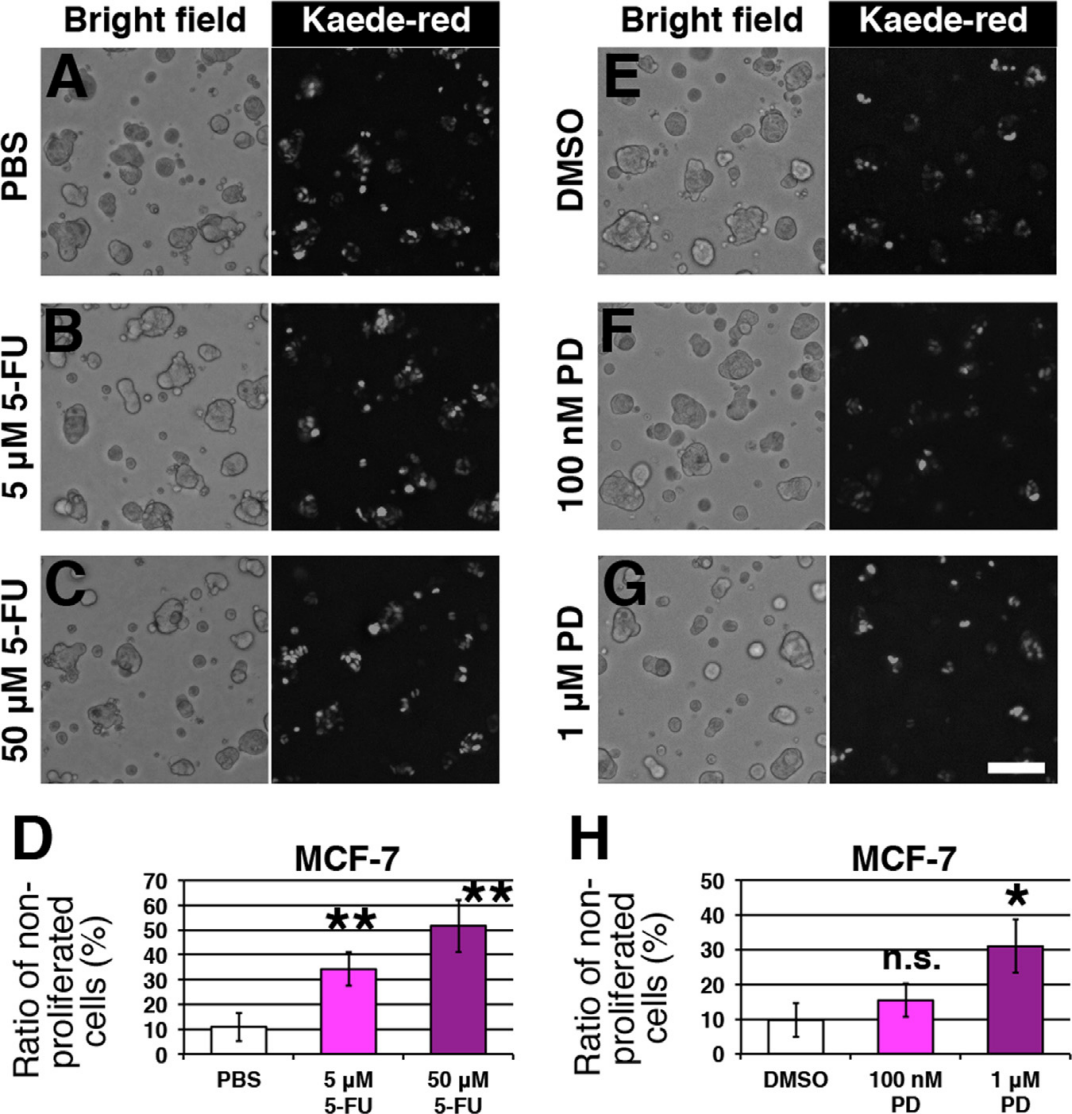
The effect of anti-cancer drugs on 3D cell growth. (A–C) Images of MCF-7 cells treated with PBS (A), 5 μM 5-FU (B), or 50 μM 5-FU (C). Bright-field and fluorescent images are shown. (D) Graph of the ratio of non-proliferated cells in the control and 5-FU treated groups (*n*=4). (E–G) Images of MCF-7 cells treated with DMSO (E), 100 nM PD0332991 (F), or 1 μM PD0332991 (G). Bright-field and fluorescent images are shown. (H) Graph of the ratio of non-proliferated cells in the control and PD0332991 treated groups (*n*=3). Bar indicates 100 μm. Error bars represent the standard deviation. PD: PD0332991, n.s.: non-significant change, *: *P*<0.05, **: *P*<0.01.

We first analyzed the effect of 5-fluorouracil (5-FU), a pyrimidine analog. Its main functions are the blocking of de novo thymidine synthesis by binding to thymidylate synthase, and inhibition of RNA processing and function by incorporation into RNA [3], [4]. After 3 days of culture, we observed no remarkable morphological changes in MCF-7 cells (Fig. 2A–C).

We analyzed the number of non-proliferated cells, and obtained a ratio of non-proliferated cells in each culture (Fig. 2D). Approximately half of the cells were non-proliferated in the presence of 50 μM 5-FU, implying that 5-FU is an effective growth inhibitor for 3D-cultured MCF-7 cells.

A cyclin-dependent kinase (CDK) 4/6 inhibitor, PD0332991, is known to affect the growth of breast cancer cells [5]. However, growth inhibition in 3D culture has not been well studied. We tested the effects of PD0332991 via our fluorescent imaging. The morphology of colonies was not altered by treatment with PD0332991 (Fig. 2E–G). As PD0332991 inhibits the function of CDK4/6, we observed an increase in the ratio of non-proliferated cells in MCF-7 cells (Fig. 2H).
