# Supplementary material for: Data of a fluorescent imaging-based analysis of anti-cancer drug effects on three-dimensional cultures of breast cancer cells
Source: Data Brief. 2015 Oct 8;5:429–33. doi: 10.1016/j.dib.2015.09.037 (PMC4773396; doi:10.1016/j.dib.2015.09.037)
Supplement: Supplementary file 1 — Supplementary material [file mmc1.docx]

**Data of a fluorescent imaging-based analysis of anti-cancer drug effects on three-dimensional cultures of breast cancer cells**

Junji Itou, Sunao Tanaka, Wenzhao Li, Yoshiaki Matsumoto, Fumiaki Sato and Masakazu Toi

Supplementary material

-A detailed procedure

**A detailed procedure of the fluorescent imaging-based assay for cell growth in 3D culture**

**<Materials>**

Medium: Phenol red-free RPMI-1640

(Life Technologies, 11835-030, Carlsbad, CA, USA)

Phenol red-free DMEM

(Wako, 044-32955, Osaka, Japan)

96-well plate: Clear-bottom 96-well plate

(BD Falcon, 353219, Franklin lakes, NJ, USA)

Matrigel: Phenol red-free Matrigel

(Corning, 356237, Bedford, MA, USA)

**<Procedure>**

1.

Liquefy Matrigel beforehand in a fridge.

Keep Matrigel on ice.

2.

Put 40 μL Matrigel at the bottom of a well of a clear bottom 96-well plate.

3.

Put the plate at 37 ℃ to solidify the Matrigel.

Wait for 30 minutes. Meanwhile, proceed with step 4 to 7.

4.

Detach cells with trypsin-EDTA.

5.

Count the number of cells.

6.

Re-suspend cells in 100 μL phenol red-free medium with serum.

(e.g. 4 x 10^4^ low proliferative cells, 2 x 10^4^ high proliferative cells)

Keep the cells on ice.

7.

Mix the cell suspension with 100 μL 10% Matrigel diluted with phenol red-free medium with serum on ice (total 200 μL, the final concentration of the Matrigel is 5%).

8.

Put the Matrigel/cell mixture into the well coated with Matrigel.

9.

Culture for one day as usual.

10.

Put the plate on the stage of an all-in-one microscope (BZ-9000) at room temperature.

11.

Irradiate cells with 340-380 nm light (excitation light for blue fluorescence) for 2.5 minutes.

12.

Take serial z-axis pictures.

Kaede-red: Excitation: 525-555 nm

Detection: 577-632 nm

Exposure time: 1 second

Bright-field: Exposure time: 1/280 seconds

*There were no saturated signals with this exposure time used under our condition.

Saturation was checked using the saturation indicator of the microscope.

Only unsaturated signals should be analyzed.

13.

Gently add 60 μL drug-containing medium with serum on the top of the culture.

The concentration of a drug in this medium is 5-fold concentrated (the final concentration is 1 fold in a total 300 μL culture).

14.

Incubate for three days as usual.

15.

Take serial z-axis pictures.

Kaede-red: Excitation: 525-555 nm

Detection: 577-632 nm

Exposure time: 1 second

Bright-field: Exposure time: 1/280 seconds
