# Supplementary material for: Data of a fluorescent imaging-based analysis of anti-cancer drug effects on three-dimensional cultures of breast cancer cells
Source: Data Brief. 2015 Oct 8;5:429–33. doi: 10.1016/j.dib.2015.09.037 (PMC4773396; doi:10.1016/j.dib.2015.09.037)
Supplement: Supplementary file 2 — Supplementary material [file mmc2.docx]

**Disclosure statement**

Junji Itou is an employee of Kyoto University’s Sponsored Research Program funded by Taiho Pharmaceutical Co., Ltd. Masakazu Toi received research funding from Taiho Pharmaceutical Co., Ltd. The funding sources had no role in the design, experiment, analysis, interpretation or writing the manuscript.
